# Comparing risk-adjusted inpatient fall rates internationally: validation of a risk-adjustment model using multicentre cross-sectional data from hospitals in Switzerland and Austria

**DOI:** 10.1186/s12913-024-10839-x

**Published:** 2024-03-13

**Authors:** Niklaus S. Bernet, Irma H. J. Everink, Sabine Hahn, Silvia Bauer, Jos M. G. A. Schols

**Affiliations:** 1 School of Health Professions, Applied Research & Development in Nursing, Bern University of Applied Sciences, Murtenstrasse 10, Bern, 3008 Switzerland; 2 Department of Health Services Research, Care and Public Health Research Institute, Maastricht University, PO BOX 616, Maastricht, 6200 MD the Netherlands; 3 Institute of Nursing Science, Medical University of Graz, Neue Stiftingtalstraße 6/P06-WEST, 8010 Graz, Austria

**Keywords:** Accidental falls, Benchmarking, Cross-sectional studies, Hospitals, Inpatients, Quality assurance, Quality of health care, Multilevel analysis, Risk adjustment

## Abstract

**Background:**

Inpatient falls in hospitals are an acknowledged indicator of quality of care. International comparisons could highlight quality improvement potential and enable cross-national learning. Key to fair cross-national comparison is the availability of a risk adjustment model validated in an international context. This study aimed to 1) ascertain that the variables of the inpatient fall risk adjustment model do not interact with country and thus can be used for risk adjustment, 2) compare the risk of falling in hospitals between Switzerland and Austria after risk adjustment.

**Methods:**

The data on inpatient falls from Swiss and Austrian acute care hospitals were collected on a single measurement day in 2017, 2018 and 2019 as part of an international multicentre cross-sectional study. Multilevel logistic regression models were used to screen for interaction effects between the patient-related fall risk factors and the countries. The risks of falling in hospital in Switzerland and in Austria were compared after applying the risk-adjustment model.

**Results:**

Data from 176 hospitals and 43,984 patients revealed an inpatient fall rate of 3.4% in Switzerland and 3.9% in Austria. Two of 15 patient-related fall risk variables showed an interaction effect with country: Patients who had fallen in the last 12 months (*OR* 1.49, 95% *CI* 1.10–2.01, *p* = 0.009) or had taken sedatives/psychotropic medication (*OR* 1.40, 95% *CI* 1.05–1.87, *p* = 0.022) had higher odds of falling in Austrian hospitals. Significantly higher odds of falling were observed in Austrian (*OR* 1.38, 95% *CI* 1.13–1.68, *p* = 0.002) compared to Swiss hospitals after applying the risk-adjustment model.

**Conclusions:**

Almost all patient-related fall risk factors in the model are suitable for a risk-adjusted cross-country comparison, as they do not interact with the countries. Further model validation with additional countries is warranted, particularly to assess the interaction of risk factors “fall in the last 12 months” and “sedatives/psychotropic medication intake” with country variable. The study underscores the crucial role of an appropriate risk-adjustment model in ensuring fair international comparisons of inpatient falls, as the risk-adjusted, as opposed to the non-risk-adjusted country comparison, indicated significantly higher odds of falling in Austrian compared to Swiss hospitals.

**Supplementary Information:**

The online version contains supplementary material available at 10.1186/s12913-024-10839-x.

## Introduction

International comparisons of quality of care outcomes have gained popularity in recent years [1, 2] and have proven to be an effective strategy for cross-national learning and the promotion of quality development [3]. By making reference values from other countries transparent, external benchmarking can highlight areas where health services are not performing as well as they could and therefore have potential for improvement [3–5].

A notable initiative in this realm, for example, is the Health Care Quality Indicators (HCQI) project led by the Organisation for Economic Co-operation and Development (OECD) [6–8]. This project was launched in 2003 to develop and publish indicators for international comparisons of health care quality, enabling benchmarking and mutual learning [4, 6, 7]. The indicators cover aspects such as acute care (e.g., 30-day mortality after acute myocardial infarction) and patient safety (e.g., post-operative complications) [6] and are published on OECD Health Statistics [9] and in the biennial OECD series “Health at a Glance” [10].

Despite the recognised potential and evidence that there are differences in quality of care outcomes within countries, but also between countries [4, 11], international comparisons are still rarely made [1]. This is predominantly attributable to the following four methodological challenges. First, despite recent developments such as defined core outcome sets [12], an internationally standardised data collection procedure is often lacking. This results in the absence of uniformly collected data [3, 13]; for example, if the information is based on primary data collection in one country or on routine data in another country [14, 15]. Second, internationally standardised definitions are frequently not applied [3, 16]. This can lead to systematic differences between countries, e.g., if a fall event is defined and calculated differently in the countries concerned, and the numerators are therefore treated differently [15]. Third, data protection regulations in different countries restrict or prevent the exchange of data across national borders and thus the use of data in an international context [17], especially when data with patient identifiers are required [1]. Fourth, in addition to data availability and data quality, the aspect of risk adjustment (or case-mix adjustment) is another challenge in the effort to enable fair and meaningful comparisons of healthcare quality outcomes (inter)nationally [1].

Differences in outcome measures between different groups, e.g., health care providers or countries, can be explained by various factors, such as differences in quality of care, in definitions, in data quality, in case mix and by chance [15]. Risk adjustment is a method that aims to control for differences in case mix. It is defined as “the process of statistically accounting for differences in patient mix that affect health care outcomes” [18]. When applying risk adjustment methods, i.e., adjusting for factors outside the direct control of health care providers, there is more certainty that differences in health care outcomes can be attributed to differences in the quality of care provided [11]. Thus, the process of risk adjustment is often paraphrased as “levelling the playing field” for a fair comparison [3, 19].

A key requirement of a risk adjustment model at an international level is that patient-related risk factors in the model have a universal character, i.e., the effects of the risk variables on the outcome should be more or less comparable in all countries studied [1, 20]. In other words, age as a patient-related risk factor should, e.g., be associated with worse outcome in country X to the same extent as in country Y. However, this required so called constant risk relationship is often not present in international comparisons, or at least unclear because it is not investigated or reported [1, 20]. By testing for interaction effects, the presence of constant risk relationships can be investigated [1, 20–22]. If no relevant interaction effects are found in the model, it indicates constant risk relationships between the patient-related risk factors and the grouping variable country, as the risk factors in each country show the same effect on the outcome.

In sum, to ensure a trustworthy international comparison of quality indicators, uniformly standardised and collected data as well as a risk adjustment model validated in an international context with regard to possible interactions must be in place. Meeting these requirements is a considerable challenge. Accordingly, few internationally comparable data and, in particular, ready-to-use risk adjustment models are available. This is especially true if, in contrast to mortality and hospitalisation rates, the quality indicators are predominantly based on primary clinical data, as in the case of the inpatient fall rate.

Inpatient fall rates in the acute care hospital setting are an internationally recognised indicator of quality of care [23, 24]. Inpatient falls are among the most frequently reported adverse events and lead to high healthcare costs, e.g., costs related to injuries and longer hospital stays [25–27]. Despite the consequences of such falls in hospitals, we are not aware of any risk-adjusted international comparisons based on clinical data. Nevertheless, an international comparison of inpatient fall rates would provide an important external reference benchmark for decision makers such as governments, health policy makers and health service managers, as well as for health professionals themselves, to assess possible national quality improvement potentials. Especially since a recently published study by Bernet et al. [28] based on data from the International Prevalence Measurement of Quality of Care (LPZ measurement) [29] showed that the performance of Swiss acute care hospitals regarding inpatient falls was very homogeneous in a national risk-adjusted comparison. The authors conclude that without a risk-adjusted external reference benchmark, there is no indication of whether lower fall rates could potentially be achieved by national quality improvements or whether high quality is already being provided compared to other countries [28]. Therefore, a risk-adjusted international comparison of inpatient fall rates can provide decision-makers at all levels with the evidence required for resource allocation. The question that needs to be answered is whether it is worth investing additional resources in inpatient fall prevention measures or whether these resources could lead to more far-reaching improvements in another area with greater quality improvement potential. To ensure that an international comparison is fair and can be used as a basis for decision-making, the inpatient fall risk adjustment model must first be validated in an international context, i.e., it must be verified whether there is a constant risk relationship between the patient-related risk factors and the countries in relation to the outcome.

As a recently developed inpatient fall risk adjustment model [28] and uniformly collected data on inpatient falls in acute care hospitals in Switzerland and Austria based on the LPZ measurement are available, we aimed to complement the Swiss data used by Bernet et al. [28] with the corresponding data from Austria in order to validate the inpatient fall risk adjustment model in an international context. Therefore, the aims of this study were:

1. to investigate whether the patient-related fall risk variables included in the inpatient fall risk adjustment model by Bernet et al. [28] show a constant risk relationship with outcome by not interacting with country, thus allowing the model to be used for a risk-adjusted comparison of the odds of falling in hospital in Switzerland and Austria.
2. to investigate whether the odds of falling in hospital in Switzerland and Austria differ after risk adjustment.

## Methods

### Study design

In this study, a data analysis was conducted based on data retrieved in the International Prevalence Measurement of Quality of Care (Landelijke Prevalentiemeting Zorgkwaliteit, LPZ measurement [29]). The LPZ measurement is an annual international multicentre cross-sectional study that captures background characteristics of patients, prevalence and prevention measures related to different quality of care indicators such as falls, pressure injuries and malnutrition in various care settings and countries. A more detailed description on the aim and procedures of the LPZ measurement can be found in Van Nie et al. [29].

This study used data on inpatient falls in Swiss and Austrian acute care hospitals (university hospitals, general hospitals and specialised clinics). The data were collected annually by the hospitals on the second Tuesday in November (14 November 2017, 13 November 2018 and 12 November 2019) using the same standardized LPZ measurement method. Other countries participating in the LPZ measurement could not be included because either no or only very few acute care hospitals participated in their measurements. The present study was conducted and reported in accordance with the RECORD (REporting of studies Conducted using Observational Routinely-collected health Data) Statement [30, 31]. The corresponding checklist used can be found in Additional file 1.

### Setting and sample

In Switzerland, all acute care hospitals that have signed up to the national quality contract (approximately 97% of Swiss acute care hospitals) are obliged to participate in the LPZ measurement. These hospitals were annually contacted by the national coordinator of Switzerland (Bern University of Applied Sciences) by e-mail and asked to register for the measurement. The national coordinator for the LPZ measurement in Austria (Medical University of Graz) annually invites all acute care hospitals by email to participate in the measurement on a voluntary basis. Participation in the LPZ measurements was subject to a fee for the hospitals. With minor differences between the countries and slight adaptations over the years, the amount the hospitals paid was calculated annually based on a fixed amount per hospital of around 500 euros and a variable amount of around 3 euros per bed. In Switzerland, however, the costs for the hospitals were covered by the Swiss Association for Quality Development in Hospitals and Clinics (ANQ).

On the measurement day of the LPZ measurement, all inpatients who were 18 years or older and from whom a verbal (Switzerland) or written (Austria) informed consent was available were included. Patients were excluded from the LPZ measurement if they were not physically present on the ward during the measurement (e.g., due to therapies or surgeries) or if informed consent was not available from the patient (e.g., comatose or terminal condition, cognitive impairment, language barrier) or their legal representative.

All patients from psychiatric or closed geriatric wards were excluded from this analysis, as these ward types participated in Austria but not in Switzerland.

### Data collection

Data collection in the LPZ measurement follows a standardised procedure, which is described in detail in the internationally defined measurement manual and is available to all people involved in the measurement in the respective language(s) of the participating country. Following the train-the-trainer principle, the national coordinators of Switzerland and Austria organised an annual training event for the measurement coordinators of each participating hospital prior to the measurement. During these trainings the measurement procedure including the inclusion and exclusion criteria, the definitions, the questionnaire and the web-based data entry programme were explained in detail. The training materials used were also made available to the hospital coordinators so that they could use them to train their clinical measurement teams.

The clinical measurement teams each consisted of two nurses (registered nurses or at least nurses who have assessment responsibilities in everyday clinical practice, one of whom works on the ward being surveyed and the other one on a different ward). During the measurement, the clinical measurement teams collected the data directly from the patient or from medical records, where permissible, according to the measurement manual and questionnaire (e.g., patient characteristics) [29].

Responses were either recorded on paper questionnaires and then transferred to the data entry programme or, alternatively, were entered directly into the data entry programme on the LPZ website. The data entry programme is designed to guide participants intuitively through the questionnaire, to display warnings for unanticipated data entries, and to prevent missing values by not allowing a questionnaire to be completed until all mandatory data have been entered. Furthermore, the transfer of the data and the data quality were determined by the operator of the LPZ data entry programme using a data profile. This involves a systematic search for error patterns in the datasets (data that should not be present; conspicuous or missing values). In addition, the national data sets are checked by the national coordinators for implausible cases, missing data and conspicuous values (e.g., very high age entries). Potentially incorrect data entries are verified together with the hospitals concerned and, where indicated, corrected by the hospitals directly in the data entry programme [32].

### Measures

The data were collected using the LPZ 2.0 instrument, which includes validated measures to assess the prevalence of various quality of care indicators, and is continuously evaluated and revised as needed by experts from an international research group [29]. The LPZ 2.0 instrument measures general patient background characteristics such as age, gender, diagnoses, etc., and quality of care indicator specific information at institution, ward and patient level. Available information that was not relevant for this study, such as information on other quality indicators (e.g., pressure injuries, malnutrition), was not included in the analysis.

The outcome variable of this study is whether or not a patient fell in hospital. Following the definition of the Kellogg International Work Group on the Prevention of Falls by the Elderly [33], a fall is defined in the LPZ measurement as any unintentional change in position that results in the client coming to rest on the ground or other lower level, regardless of the reason [28, 29]. Inpatient falls were retrospectively assessed with the question: “Has the client fallen in the last 30 days in this institution?” (yes/no). All other variables considered in this study are patient-related fall risk factors that were identified as relevant based on the inpatient fall risk adjustment model developed by Bernet et al. [28]. Table 1 gives an overview of the variables used in the present study and in the inpatient fall risk adjustment model of Bernet et al. [28].

**Table 1:** Variables included in the inpatient fall risk adjustment model and therefore in the present study

**Outcome variable**	**Answer options**
Has the client fallen in the last 30 days in this institution	no [0] yes [1]
**Characteristics/patient-related fall risk factors**	**Answer options**
Age (in years) / Age groups	Scale / 18–64 years [0] 65–74 years [1] 75–84 years [2] ≥ 85 years [3]
Sex	male [0] female [1]
Care Dependency Scale (CDS)	care independent (70–75) [0] to a great extent independent (60–69) [1] partially dependent (45–59) [2] to a great extent dependent (25–44) [3] completely dependent (15–24) [4]
Fall in the last 12 months before hospital admission	no [0] yes [1]
Sedative/psychotropic medication intake	no [0] yes [1]
Surgical procedure within 14 days prior to measurement	no [0] yes [1]
ICD-10—Mental and behavioural disorders [yes]	no [0] yes [1]
ICD-10—Neoplasms	no [0] yes [1]
ICD-10—Diseases of the blood and blood-forming organs	no [0] yes [1]
ICD-10—Certain infectious and parasitic diseases	no [0] yes [1]
ICD-10—Factors influencing health status	no [0] yes [1]
ICD-10—Diseases of the nervous system	no [0] yes [1]
ICD-10—Endocrine, nutritional and metabolic diseases	no [0] yes [1]
ICD-10—Diseases of the musculoskeletal system	no [0] yes [1]
ICD-10—Diseases of the ear [yes]	no [0] yes [1]

The Care Dependency Scale consists of 15 items (e.g., food and drink, continence, mobility). The sum score ranges from 15 to 75 points [34, 35] and can be categorized into five categories: Completely dependent on care from others, to a great extent dependent, partially dependent, to a great extent independent and care independent [36, 37]

*ICD-10* International statistical classification of diseases and related health problems 10th revision [38]

### Data analysis

The data analysis was divided into seven main steps, which are described in detail below (see also Additional file 2 for a summarised overview of the main analysis steps).

First, the complete Swiss dataset used for the development of the inpatient fall risk adjustment model in the study by Bernet et al. [28] was supplemented with the complete Austrian datasets of the 2017, 2018 and 2019 measurements.

Second, to obtain comparable data sets between Switzerland and Austria and to promote accurate estimates in the context of a multilevel model, all hospitals with fewer than 50 participants over the three measurement years were excluded from the analysis, analogous to the procedure described in Bernet et al. [28].

Thirdly, for sample description, the number of hospitals and the patient-related fall risk factors according to Table 1 were described with frequencies and percentages. In addition, Pearson's chi-square test was used to examine whether patient-related fall risk factors differed significantly between the two countries. As a baseline model to illustrate a non-risk-adjusted comparison of inpatient fall rates across Switzerland and Austria, a two-level random intercept logistic regression model was calculated, with hospitals modelled as a random effect and country as a fixed effect. The corresponding odds ratio (*OR*) and 95% confidence interval (95% *CI*) were reported.

Fourth, to answer the question of whether there are interaction effects between the patient-related fall risk factors of the inpatient fall risk adjustment model of Bernet et al. [28] and the country, the inpatient fall risk adjustment model was applied to the merged Swiss and Austrian data within the framework of a two-level random intercept logistic regression model. In this, the hospitals are modelled as random effect and the patient-related fall risk factors according to Table 1 as fixed effects. In addition, the interaction terms between the patient-related fall risk factors and the country are considered as fixed effects in the model (hereafter denoted as “Model 1”). Following Moger and Peltola [1], Mohammed et al. [20], Goodacre et al. [21] the interaction terms were used to screen whether the proposed inpatient fall risk adjustment model fulfils the basic requirement of including universal fall risk factors, i.e., whether they are constantly associated with a higher or lower inpatient fall risk regardless of the country and can therefore safely be used for a risk-adjusted international comparison of inpatient falls in Switzerland and Austria. If interaction effects are present, they may indicate a non-constant risk relationship and thus the danger of the so-called constant risk-fallacy [22]. The constant risk fallacy means that, contrary to the intention of risk adjustment to reduce bias in comparisons, bias may instead increase [1, 20, 22]. Significant interaction effects, however, do not provide any information as to the causes of the non-constant risk relationships found, and consequently whether these are problematic for a risk-adjusted country comparison (or whether the non-constant risk relationships are instead due to differences in the quality of care provided). If there are significant interaction effects, however, this is an indication that further attention is needed. The inpatient fall risk adjustment model did not converge initially, which is not uncommon in complex modelling situations but may lead to untrustworthy model estimates [39]. As the convergence issue was related to the continuous variable “age” in the model, we decided to transform the variable into a categorical variable in order to reduce model complexity. Following Heikkilä et al. [40] and Thomann et al. [41] the subsequent four age categories were created: 18–64 years, 65–74 years, 75–84 years and older than 84 years. By introducing “age” as a categorical variable in the inpatient fall risk adjustment model, the convergence problems could be solved. To test for a possible measurement year effect, we recalculated “Model 1” by including the measurement year as a control variable. The measurement year was not significant in the model and the Akaike Information Criterion (AIC) value was higher than in the initial “Model 1”. Therefore, only the initial model (“Model 1”) without the control variable measurement year was subsequently reported.

Fifth, in the context of a model development process, a reduced two-level random intercept logistic regression model (hereafter denoted as “Model 2”) was calculated to capture the main interaction effects. In “Model 2”, in addition to the patient-related fall risk factors, only those 2 × 2 interaction terms were included in the model that had a *p*-value smaller than 0.16 in “Model 1”. This less stringent threshold (compared to a *p*-value < 0.05) was chosen so that important interaction effects would not be overlooked due to lack of power or stochastic variability and because this value roughly corresponds to a variable selection based on the AIC [42]. In addition, to decide whether the interaction effects of the variables age and CDS, which have several factor levels, are relevant overall, AIC was used to compare “Model 1” with a model without the interaction term Age*Country or CDS*Country. If the AIC value in the model without the corresponding interaction term was lower than in “Model 1”, the corresponding interaction term was not taken into account in “Model 2”.

Sixth, to clarify whether the inpatient fall risk adjustment model accounts equally for key patient-related fall risk variables in Switzerland and Austria, we applied the model separately to the Swiss and Austrian datasets. We subsequently compared the performance of the model in terms of discrimination using receiver operating characteristic (ROC) curve analysis.

Seventh, to answer the question of whether the risk of falling in hospital in Switzerland and Austria differ after risk adjustment, we applied the inpatient fall risk adjustment model of Bernet et al. [28] to the merged Swiss and Austrian data within the framework of a two-level random intercept logistic regression model. The hospitals are modelled as random effect and the patient-related fall risk factors according to Table 1 as fixed effects.

Data preparation and descriptive analyses were carried out with IBM© SPSS© Statistics (version 28). The multilevel logistic regression models and the ROC curves were built and analysed with the statistics programme R, version 4.1.0 [43], and the packages *lme4* [44], *sjplot* [45], *effects* [46, 47] and *pROC* [48]. The statistical significance level was set at *p*-value below 0.05 in all analyses.

## Results

A total of 176 hospitals with 43,984 patients who participated in the 2017, 2018 and 2019 measurements in Switzerland and Austria were included in the analysis. Of these, 138 (78.4%) hospitals were from Switzerland and 38 (21.6%) from Austria. Thus, the majority of the participants were hospitalised in Swiss hospitals (81.8%, *n* = 35,998). The distribution of participants across hospital types was similar in the two countries, with the largest proportion of participants coming from general hospitals (Switzerland 73.9%,* n* = 26,590; Austria 70.3%, *n* = 5,611), followed by university hospitals (Switzerland 19.4%, *n* = 6,982; Austria 26.1%, *n* = 2,088) and specialised hospitals (Switzerland 6.7%, *n* = 2,426; Austria 3.6%, *n* = 287). Almost half of the participants were female (49.5%, *n* = 21,787), aged between 18–74 years (60.9%, *n* = 26,770) and care independent according to the CDS (55.2%, *n* = 24,273). Nearly one third of participants had experienced a fall in the last 12 months prior to hospital admission (29.7%, *n* = 13,073). A similar proportion had been exposed to current (in the past 24 h), periodic (irregular) and/or continuous (regular) intake of sedatives or psychotropic medication (35.0%, *n* = 15,377). Further, 40.8% (*n* = 17,954) of all participants had undergone a surgical procedure in the 14 days prior to the measurement. The participants were most frequently affected by a diagnosis from the ICD-10 group “Diseases of the musculoskeletal system” (38.2%, *n* = 16,823), followed by “Endocrine, nutritional and metabolic diseases” (34.9%, *n* = 15,350) and “Neoplasms” (21.5%, *n* = 9,452).

When comparing the patient-related fall risk factors between the two countries (see Table 2 and Additional file 3 for a graphic overview), a significant difference was found in almost all variables. The most prominent differences were the higher proportion of care independent participants in Austria, as well as a lower proportion of participants in Austria who had had a fall in the last 12 months. Also significantly fewer participants in Austria were taking sedatives and/or psychotropic medication and had had a surgical procedure in the 14 days prior to the measurement. Of the nine ICD-10 diagnosis groups reported, the percentages of Austrian participants with these diagnoses were significantly lower than in Switzerland for seven, and significantly higher for one of the ICD-10 diagnosis groups.

**Table 2:** Descriptive and bivariate comparison of patient-related fall risk factors between Switzerland and Austria

	**Switzerland (*n* = 35,998)**		**Austria (*n* = 7,986)**		
**Patient-related fall risk factors**	*n*	%	*n*	%	*p*-value
Sex [female]	17,669	49.1	4,118	51.6	**< 0.001**
Age groups					
18–64 years	13,738	38.2	2,981	37.3	**< 0.001**
65–74 years	8,186	22.7	1,865	23.4	
75–84 years	8,913	24.8	2,133	26.7	
≥ 85 years	5,161	14.3	1,007	12.6	
Care dependency (CDS)					
care independent [70–75]	19,247	53.5	5,026	62.9	**< 0.001**
to a great extent independent [60–69]	8,807	24.5	1,320	16.5	
partially dependent [45–59]	5,218	14.5	849	10.6	
to a great extent dependent [25–44]	2,083	5.8	516	6.5	
completely dependent [15–24]	643	1.8	275	3.4	
Fall in the last 12 months [yes]	11,131	30.9	1,942	24.3	**< 0.001**
Sedative/psychotropic medications intake [yes]	12,928	35.9	2,449	30.7	**< 0.001**
Surgical procedure within 14 days prior to measurement [yes]	15,885	44.1	2,069	25.9	**< 0.001**
ICD-10—Mental and behavioural disorders [yes]	6,932	19.3	1,093	13.7	**< 0.001**
ICD-10—Neoplasms [yes]	7,895	21.9	1,557	19.5	**< 0.001**
ICD-10—Diseases of the blood and blood-forming organs [yes]	6,074	16.9	709	8.9	**< 0.001**
ICD-10—Certain infectious and parasitic diseases [yes]	4,788	13.3	585	7.3	**< 0.001**
ICD-10—Factors influencing health status [yes]	2,513	7.0	691	8.7	**< 0.001**
ICD-10—Diseases of the nervous system [yes]	5,172	14.4	1,072	13.4	**0.029**
ICD-10—Endocrine, nutritional and metabolic diseases [yes]	12,617	35.0	2,733	34.2	0.161
ICD-10—Diseases of the musculoskeletal system [yes]	14,626	40.6	2,197	27.5	**< 0.001**
ICD-10—Diseases of the ear [yes]	959	2.7	145	1.8	**< 0.001**

*CDS* Care dependency scale, *ICD-10* International statistical classification of diseases and related health problems 10th revision

Significant differences between countries according to the Pearson's chi-square test results are highlighted in bold

The outcome analyses showed that a total of 1,551 participants experienced an inpatient fall. This corresponds to an overall inpatient fall rate of 3.5%. The inpatient fall rate by country was 3.4% (*n* = 1,239) in Switzerland and 3.9% (*n* = 312) in Austria. The non-risk-adjusted country comparison (baseline model that, in addition to the random effect, only takes the country variable into account as a fixed effect) showed that the odds of falling in hospital do not differ between Switzerland and Austria (*OR* 0.99, 95% *CI* 0.77–1.28, *p* = 0.961).

### Interactions between patient-related fall risk factors and country

In “Model 1” (see Table 3 for a description) the interaction effects between the 15 patient-related fall risk factors and the two countries were taken into account. There was a significant interaction effect between the variable “country” and “having a fall in the last 12 months before hospital admission” on the outcome measure inpatient falls (*OR* 1.33, 95% *CI* 1.00–1.76, *p* = 0.049). In addition, two interaction effects were found with a *p*-value of less than 0.16. The effect of sedatives/psychotropic medication intake (*OR* 1.31, 95% *CI* 0.98–1.74, *p* = 0.067) as well as the effect of having a surgical procedure (*OR* 0.70, 95% *CI* 0.48–1.03, *p* = 0.069) on the risk of falling in hospital differs between Swiss and Austrian patients. All other interaction terms exceed a *p*-value of 0.16. Therefore, the odds of experiencing an inpatient fall while, e.g., affected by a mental or behavioural disorder is not dependent on being a patient in Switzerland or Austria (*OR* 0.86, 95% *CI* 0.63–1.18, *p* = 0.349). In other words, most of the patient-related fall risk variables in the model show a constant risk relationship with the outcome inpatient fall, regardless of the country.

**Table 3:** Description of “Model 1” and “Model 2”^1^

	**Model 1**			**Model 2**		
*Predictors*	*OR*	*95% CI*	*p-value*	*OR*	*95% CI*	*p-value*
CDS [care independent (70–75)]	Ref			Ref		
CDS [to a great extent independent (60–69)]	**2.20**	**1.86 – 2.61**	**< 0.001**	**2.24**	**1.93 – 2.61**	**< 0.001**
CDS [partially dependent (45–59)]	**3.08**	**2.58 – 3.68**	**< 0.001**	**2.97**	**2.53 – 3.48**	**< 0.001**
CDS [to a great extent dependent (25–44)]	**3.55**	**2.88 – 4.38**	**< 0.001**	**3.31**	**2.75 – 3.99**	**< 0.001**
CDS [completely dependent (15–24)]	**1.95**	**1.34 – 2.83**	**0.001**	**1.96**	**1.43 – 2.68**	**< 0.001**
Fall in the last 12 months [yes]	**2.17**	**1.91 – 2.46**	**< 0.001**	**2.20**	**1.95 – 2.50**	**< 0.001**
Sedative/psychotropic medication intake [yes]	**1.75**	**1.54 – 1.98**	**< 0.001**	**1.78**	**1.57 – 2.02**	**< 0.001**
Age [18–64 years]	Ref			Ref		
Age [65–74 years]	**1.35**	**1.12 – 1.62**	**0.002**	**1.45**	**1.23 – 1.71**	**< 0.001**
Age [75–84 years]	**1.60**	**1.34 – 1.90**	**< 0.001**	**1.59**	**1.36 – 1.86**	**< 0.001**
Age [≥ 85 years]	**1.55**	**1.28 – 1.88**	** < 0.001**	**1.56**	**1.31 – 1.85**	**< 0.001**
ICD-10—Mental and Behavioural disorders [yes]	**1.55**	**1.36 – 1.77**	** < 0.001**	**1.52**	**1.35 – 1.71**	**< 0.001**
ICD-10—Neoplasms [yes]	**1.26**	**1.10 – 1.44**	**0.001**	**1.21**	**1.07 – 1.37**	**0.002**
ICD-10—Diseases of the blood and blood-forming organs [yes]	**1.23**	**1.07 – 1.41**	**0.003**	**1.25**	**1.10 – 1.42**	**0.001**
ICD-10—Certain infectious and parasitic diseases [yes]	**1.19**	**1.02 – 1.38**	**0.029**	1.15	1.00 – 1.33	0.055
ICD-10—Factors influencing health status [yes]	1.21	0.99 – 1.46	0.059	**1.22**	**1.03 – 1.45**	**0.023**
ICD-10—Diseases of the nervous system [yes]	1.14	0.99 – 1.32	0.068	**1.14**	**1.00 – 1.30**	**0.045**
ICD-10—Endocrine, nutritional and metabolic diseases [yes]	**1.13**	**1.00 – 1.28**	**0.047**	**1.12**	**1.01 – 1.25**	**0.035**
ICD-10—Diseases of the musculoskeletal system [yes]	0.91	0.80 – 1.03	0.116	0.89	0.80 – 1.00	0.050
Surgical procedure within 14 days prior to measurement [yes]	**0.82**	**0.72 – 0.93**	**0.003**	**0.81**	**0.71 – 0.92**	**0.002**
Sex [female]	**0.79**	**0.70 – 0.89**	**< 0.001**	**0.76**	**0.68 – 0.85**	**< 0.001**
ICD-10—Diseases of the ear [yes]	**0.69**	**0.48 – 0.99**	**0.043**	**0.69**	**0.49 – 0.97**	**0.031**
Country [Austria]	1.36	0.88 – 2.12	0.168	1.19	0.89 – 1.60	0.250
CDS [to a great extent independent (60–69)]:Country [Austria]	1.14	0.79 – 1.64	0.475^2^			
CDS [partially dependent (45–59)]:Country [Austria]	0.82	0.55 – 1.22	0.326^2^			
CDS [to a great extent dependent (25–44)]:Country [Austria]	0.72	0.46 – 1.15	0.172^2^			
CDS [completely dependent (15–24)]:Country [Austria]	1.04	0.52 – 2.06	0.920^2^			
Fall in the last 12 months [yes]:Country [Austria]	**1.33**	**1.00 – 1.76**	**0.049**	1.25	0.96 – 1.64	0.102
Sedative/psychotropic medication intake [yes]:Country [Austria]	1.31	0.98 – 1.74	0.067	1.18	0.90 – 1.55	0.230
Age [65–74 years]:Country [Austria]	1.38	0.92 – 2.06	0.122^3^			
Age [75–84 years]:Country [Austria]	0.97	0.65 – 1.44	0.880^3^			
Age [= 85 years]:Country [Austria]	1.02	0.65 – 1.60	0.926^3^			
ICD-10—Mental and Behavioural disorders [yes]:Country [Austria]	0.86	0.63 – 1.18	0.349			
ICD-10—Neoplasms [yes]:Country [Austria]	0.81	0.57 – 1.13	0.208			
ICD-10—Diseases of the blood and blood-forming organs [yes]:Country [Austria]	1.08	0.74 – 1.56	0.700			
ICD-10—Certain infectious and parasitic diseases [yes]:Country [Austria]	0.80	0.51 – 1.25	0.322			
ICD-10—Factors influencing health status [yes]:Country [Austria]	1.03	0.68 – 1.58	0.878			
ICD-10—Diseases of the nervous system [yes]:Country [Austria]	0.98	0.70 – 1.38	0.904			
ICD-10—Endocrine, nutritional and metabolic diseases [yes]:Country [Austria]	0.94	0.72 – 1.23	0.641			
ICD-10—Diseases of the musculoskeletal system [yes]:Country [Austria]	0.93	0.69 – 1.25	0.623			
Surgical procedure within 14 days prior to measurement [yes]:Country [Austria]	0.70	0.48 – 1.03	0.069	0.73	0.50 – 1.07	0.104
Sex [female]:Country [Austria]	0.83	0.63 – 1.09	0.178			
ICD-10—Diseases of the ear [yes]:Country [Austria]	0.92	0.31 – 2.75	0.882			
**Random Effects**						
σ^2^ [residual variance]	3.29			3.29		
τ_00_ [variability in hospital intercepts]	0.09			0.09		
N [hospitals]	176			176		
Observations	43,984			43,984		
AIC	11,871			11,855		

*CDS* Care dependency scale, *ICD-10* International statistical classification of diseases and related health problems 10th revision, *Ref*. reference category, significant *OR* are highlighted in bold

^1^ “Model 1” describes the inpatient fall risk adjustment model including all interaction terms regarding patient-related fall risk factors and country and “Model 2” describes the inpatient fall risk adjustment model including the relevant interaction effects found in “Model 1”

^2^ “Model 1” without the interaction term CDS*Country revealed a lower AIC value of 11,869. The interaction term CDS*Country is therefore not included in “Model 2”

^3^ “Model 1” without the interaction term Age*Country revealed a lower AIC value of 11,870. The interaction term Age*Country is therefore not included in “Model 2”

The comparison of “Model 1” and “Model 2” shows that the reduced “Model 2” fits the data better with an AIC value of 11,855 compared with a value of 11,871 for “Model 1” (see Table 3). The three interaction effects remaining in “Model 2” are not statistically significant. If the individual subgroups are examined in detail, both significant and non-significant differences between the countries become apparent. Swiss and Austrian patients without a fall in the last 12 months, without sedative/psychotropic medication or without surgery have no significantly higher or lower odds of falling in hospital (*OR* 1.19, 95% *CI* 0.89–1.60, *p* = 0.250). However, patients who have fallen in the last 12 months (*OR* 1.49,^1^ 95% *CI* 1.10–2.01, *p* = 0.009) or take sedatives/psychotropic medication (*OR* 1.40^1^, 95% *CI* 1.05–1.87, *p* = 0.022) have significantly higher odds of falling in hospital in Austria than in Switzerland. Although not statistically significant, the risk of falling is lower for Austrian patients with surgery (*OR* 0.87^1^, 95% *CI* 0.58–1.31, *p* = 0.508) than for Swiss patients.

### Discriminatory performance of the inpatient fall risk adjustment model

**Fig. 1:**
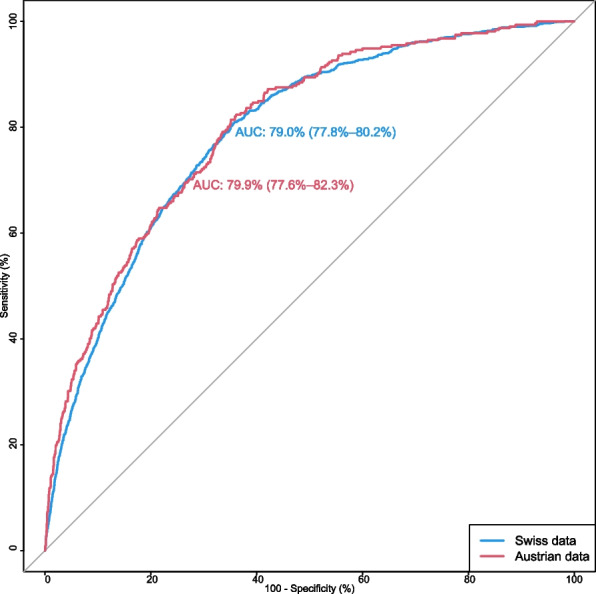
ROC curves of the inpatient fall risk adjustment model based on Swiss and Austrian data

The inpatient fall risk adjustment model was originally developed based on the Swiss data. Applying the model to this data set showed an area under the curve (AUC) of 79.0% (95% *CI* 77.8–80.2). In comparison (see Fig. 1), the AUC based on the Austrian data set showed a value of 79.9% (95% *CI* 77.6–82.3) and thus does not differ significantly (*p* = 0.505). The covariates included in the inpatient fall risk adjustment model are equally able to predict a fall in hospital in both countries.

After controlling for differences in patient-related fall risk factors between the two countries by applying the inpatient fall risk adjustment model, a statistically significant country difference in the odds of falling in hospital emerges (Table 4). After risk adjustment, Austrian patients show significantly higher odds of falling in hospital than do Swiss patients (*OR* 1.38, 95% *CI* 1.13–1.68, *p* = 0.002).

**Table 4:** Risk-adjusted comparison of the odds of falling in hospital in Switzerland or Austria

*Predictors*	*OR*	*95% CI*	*p-value*
CDS [care independent (70–75)]	Ref		
CDS [to a great extent independent (60–69)]	**2.26**	**1.94 – 2.62**	**< 0.001**
CDS [partially dependent (45–59)]	**2.98**	**2.54 – 3.49**	**< 0.001**
CDS [to a great extent dependent (25–44)]	**3.32**	**2.75 – 4.00**	**< 0.001**
CDS [completely dependent (15–24)]	**1.97**	**1.44 – 2.69**	** < 0.001**
Fall in the last 12 months [yes]	**2.31**	**2.06 – 2.58**	**< 0.001**
Sedative/psychotropic medication intake [yes]	**1.84**	**1.64 – 2.07**	**< 0.001**
Age [18–64 years]	Ref		
Age [65–74 years]	**1.45**	**1.23 – 1.71**	**< 0.001**
Age [75–84 years]	**1.59**	**1.36 – 1.86**	**< 0.001**
Age [≥ 85 years]	**1.56**	**1.31 – 1.85**	**< 0.001**
ICD-10—Mental and Behavioural disorders [yes]	**1.51**	**1.34 – 1.70**	**< 0.001**
ICD-10—Neoplasms [yes]	**1.21**	**1.07 – 1.37**	**0.003**
ICD-10—Diseases of the blood and blood-forming organs [yes]	**1.25**	**1.10 – 1.42**	**0.001**
ICD-10—Certain infectious and parasitic diseases [yes]	1.15	0.99 – 1.32	0.060
ICD-10—Factors influencing health status [yes]	**1.22**	**1.03 – 1.45**	**0.021**
ICD-10—Diseases of the nervous system [yes]	**1.14**	**1.00 – 1.30**	**0.049**
ICD-10—Endocrine, nutritional and metabolic diseases [yes]	**1.12**	**1.01 – 1.25**	**0.040**
ICD-10—Diseases of the musculoskeletal system [yes]	0.90	0.80 – 1.00	0.053
Surgical procedure within 14 days prior to measurement [yes]	**0.78**	**0.69 – 0.88**	**< 0.001**
Sex [female]	**0.76**	**0.68 – 0.85**	**< 0.001**
ICD-10—Diseases of the ear [yes]	**0.68**	**0.49 – 0.96**	**0.029**
Country [Austria]	**1.38**	**1.13 – 1.68**	**0.002**
**Random Effects**			
σ^2^ [residual variance]	3.29		
τ_00_ [variability in hospital intercepts]	0.09		
N [hospitals]	176		
Observations	43,984		
AIC	11,856.5		

*CDS* Care dependency scale, *ICD-10* International statistical classification of diseases and related health problems 10th revision, significant *OR* are highlighted in bold

## Discussion

In this study, we investigated the extent to which the effects of the patient-related fall risk factors used for risk adjustment in connection with inpatient falls developed with Swiss data remained constant when applied to an external Austrian data set, thus allowing them to be used for a risk-adjusted comparison of inpatient falls between Switzerland and Austria. Of the total of 15 patient-related fall risk variables in the model, we could not find any evidence of relevant interaction effects with the grouping variable “country” for 12 variables. We therefore conclude that the majority of risk factors used in the inpatient fall risk adjustment model are associated with inpatient falls regardless of country, which indicates that the risk factors can safely be used in the risk adjustment model to compare the inpatient falls of Austria and Switzerland [22]. In addition, we demonstrated that the risk of falling in hospital in Switzerland and Austria differs statistically significantly after risk adjustment.

Three of the patient-related fall risk variables included in the models, namely “fall in the last 12 months”, “sedatives/psychotropic medication intake” and “surgical procedure within 14 days prior to measurement”, showed, at least in part, evidence for a non-constant relationship. Even though all three interaction effects in “Model 2” (reduced model, controlling only for relevant country-specific interaction effects identified in “Model 1”) exceeded the statistical significance level of a *p*-value smaller than 0.05, significant subgroup differences were found between Switzerland and Austria for two of the risk variables. Specifically, patients in Austria with a fall in the last 12 months or who take sedative/psychotropic medication have a higher risk of falling in hospital than have Swiss patients.

In general, significant interactions and thus non-constant risk relationships according to Moger and Peltola [1], Mohammed et al. [20], Goodacre et al. [21] may be due to the following scenarios: (a) a non-uniform measurement procedure is used (the risk variable is recorded or collected differently in the two countries, which may result in underreporting in one country, for example); (b) the risk variable actually reflects a different risk association with outcome in different patient populations (e.g., the risk of inpatient falls is lower in a group of completely immobile patients who have fallen in the last 12 months than in a group of mobile patients who have fallen in the last 12 months); c) the quality of care differs between countries in relation to patients with or without risk (e.g., patients with a fall in the last 12 months may or may not receive standard fall prevention measures in one country).

In the context of a fair international comparison, scenarios a and b are problematic because the risk adjustment model assumes a constant risk relationship between the risk variables and the outcome in different populations, which may not actually be the case. This can lead to a risk-adjusted comparison being unintentionally more biased than would be the case with a purely descriptive comparison [20, 22, 49]. Scenario c is unproblematic, as the interaction effect reflects the actual differences in the quality of care provided in the two countries and not a non-constant risk relationship per se. Thus, by assuming a constant risk relationship in the risk adjustment process, the country which, for example, implements standardised fall prevention measures (quality of care) for high-risk patients (falls in the last 12 months before hospital admission) and thus achieves lower fall rates in this group, is correctly attributed with a higher quality of care.

Because there is no standard procedure or solution for identifying the underlying reasons for an interaction, and because reasons are usually not preclusive [1, 20], we can only speculate about the reasons for the subgroup differences found in our study and thus whether and, if so, to what extent these are problematic for a risk-adjusted comparison of inpatient falls in Switzerland and Austria. Since the measurement in both countries is conducted using the same method, i.e., according to a highly standardised procedure, a non-uniform measurement procedure is rather unlikely to be the reason. Furthermore, the risk factors “fall in the last 12 months” and “sedatives/psychotropic medication intake” are consistently described in the international literature as central risk factors for inpatient falls in various studies done in different contexts as well as in international guidelines [28, 50–55]. It can be assumed that they are relatively consistently associated with a higher risk of falling in hospital, regardless of the country or the population being compared. The subgroup differences found in our study can thus possibly be explained by country-specific differences in fall prevention practice (quality of care) in these subgroups and not by non-constant risk relationships.

In order to be able to substantiate or refute the speculations, a more comprehensive application of the risk adjustment model to international data sets from several countries is recommended. A basic prerequisite is that the hospitals and countries included in the international comparison ensure and adhere to a uniform approach to data collection as well as the precise definition and operationalisation of key variables in order to exclude or at least minimise systematic bias in the underlying data [3, 13, 14, 16].

Interestingly, our study also showed that, based on our sample, the non-risk-adjusted country comparison shows no difference and the comparison adjusted for patient-related fall risk factors shows a significant difference in the odds of falling in hospital between countries. The conclusions that one might draw based on the non-risk-adjusted results are in some ways the opposite of those based on the risk-adjusted results. This highlights the potential pitfalls of a non-risk-adjusted comparison of outcomes across countries, as in a worst-case scenario this could potentially lead to misguided inactivity or actionism by national-level decision-makers and healthcare providers, but also by healthcare workers. Based on the non-risk-adjusted results, it is conceivable that inpatient fall prevention in Austria would be defined as a non-priority area for quality improvement, therefore shifting the allocation of corresponding resources to other areas, which in turn would not be available for improving fall prevention, especially given the limited resources in the health care system.

It should be noted that the results of an international comparison, whether risk-adjusted or not, should always be contextualised. International comparisons are useful for identifying signals of quality deficits. They do not usually provide an answer to the question of how to improve the quality of care, but offer an evidence-based starting point for a more in-depth analysis to better understand the results found and to stimulate a debate on quality of care and cross-national learning [3, 56]. As a starting point for mutual cross-national learning and to inform clinical practice, it would be interesting for Austrian healthcare institutions to examine what is done differently in practice in Switzerland. In particular, it would be interesting for nurses to analyse which preventive nursing measures are routinely implemented in clinical practice in Switzerland to prevent falls in patients who have had a fall in the last 12 months or who are taking sedative/psychotropic medication.

### Strength and limitations

A strength of our study is the first validation and application of an inpatient fall risk adjustment model based on a large sample from two geographically close European countries. In contrast to other international comparisons, which are usually based on administrative data and therefore on data of potentially limited quality, e.g., due to non-uniform coding practices [57], we were able to draw on standardised and uniformly collected primary data. This aspect in particular is highly relevant because, as described in the introduction, differences in an outcome between groups can also be caused by differences in data collection and, consequently, data quality. This possibility could be largely eliminated in our study through the use of uniformly collected data.

It should be noted, however, that in contrast to hospitals in Switzerland, which were obliged to participate in the measurement, and the costs were covered by ANQ, the hospitals in Austria had to pay a fee to participate. A selection bias in Austria needs to be assumed. It is likely that only those hospitals that consider the measurement of quality indicators to be important and are sensitised to it participated in the measurement and invested money accordingly. The differences found in the sample characteristics of the two countries could be an indication of a possible selection bias. In this context, it would have been interesting to compare the ward types participating in the measurement per country, as an over- or under-representation of certain patient characteristics could be related to an over- or under-representation of certain ward types in the sample. Unfortunately, not all ward types were defined uniformly in Switzerland and Austria, and the individual wards of a hospital sometimes changed their ward type over the measurement years, so that it was not always possible to assign an individual ward to a ward type with certainty. The voluntary nature of participation in the measurement in Austria could also be the reason for the differences in sample size between the two countries. For the risk-adjusted comparison of inpatient fall rates, this is in principle unproblematic, as we were able to show that the effects of the patient-related risk variables are largely constant in the two countries. Accordingly, it is irrelevant how many cases a country contributes to the overall data set [1]. However, due to the before mentioned possible selection bias, the data on which the risk-adjusted comparison of inpatient fall rates is based may not be completely representative for Austria and therefore the generalisability of the results to the national level is limited.

Additionally, in order to increase the stability and the estimates of the risk adjustment model, it would have been desirable to collect data over a longer time period instead of cross-sectional data. This is hardly feasible in terms of resources within the framework of primary data collection at the national level. We tried to take this into account by pooling the three measurement points. Although the LPZ measurement is also carried out in other countries such as the Netherlands and Turkey, it was not possible to include these countries in the analyses due to too few participating hospitals and correspondingly fewer participating patients in these countries. If data from several countries were available in the future, this would favour further external validation of the inpatient fall risk adjustment model.

## Conclusions

In our study, we presented first results on the applicability of the inpatient fall risk adjustment model for international comparison. The majority of the patient-related fall risk factors considered in the model can be used for the risk adjustment of inpatient fall rates at the international level, as hardly any significant interactions could be identified. Moreover, the patient-related fall risk factors considered in the inpatient fall risk adjustment model are equally able to predict a fall in hospital, regardless of the country. However, further investigation in a broader international context involving several countries is warranted. In particular, it is recommended to check to what extent the fall risk factors “fall in the last 12 months” and “sedatives/psychotropic medication intake” interact with the grouping variable country. Furthermore, we were able to show that the results differ when the inpatient fall risk is compared in Switzerland and Austria without and with adjustment for patient-related risk factors. The non-risk-adjusted results imply a non-significant difference in national fall rates between the two countries, but the risk-adjusted comparison suggests a significant one. This illustrates that the application of a risk adjustment model can significantly alter foundations for decision-making and thus may lead to different conclusions by decision-makers regarding quality improvement potential. We conclude that our inpatient fall risk adjustment model enables a risk-adjusted international comparison of inpatient fall rates and thus contributes significantly to achieving the goal of a fair comparison. Also, recognising the imperfection of any risk-adjustment model, we recommend that decision-makers consider risk-adjusted international comparisons whenever possible and available, ideally in addition to national results over time, to incorporate the most comprehensive and robust information possible in assessing potential for improvement in the area of inpatient falls.

### Supplementary Information

**Supplementary Material 1. **

**Supplementary Material 2. **

**Supplementary Material 3. **

## Data Availability

The Swiss and Austrian data that support the findings of this study are available from the Swiss National Association for Quality Development in Hospitals and Clinics (ANQ) and the Department of Nursing Science at the Medical University of Graz, respectively, but restrictions apply to the availability of these data, which were used under license for the current study, and so are not publicly available. Therefore, the data are available upon reasonable request and with the permission of the Swiss Association for Quality Development in Hospitals and Clinics (ANQ) and the Department of Nursing Science at the Medical University of Graz from the corresponding author.

## Abbreviations

95% CI: 95% Confidence interval
AIC: Akaike Information Criterion
AUC: Area Under the Curve
CDS: Care Dependency Scale
ICD-10: International Statistical Classification of Diseases and Related Health Problems 10th Revision
LPZ: National Prevalence Measurement of Quality of Care (in Dutch: Landelijke Prevalentiemeting Zorgkwaliteit)
OR: Odds Ratio
ROC: Receiver Operating Characteristics

## Footnotes

1 The odds ratios of subgroup comparisons, which are not shown in the tables due to the reference category used, were calculated and reported in the text as follows: OR_Country_ multiplied by OR_Interaction term_ = OR_reported in text._
