# Supplementary material for: Comparing risk-adjusted inpatient fall rates internationally: validation of a risk-adjustment model using multicentre cross-sectional data from hospitals in Switzerland and Austria
Source: BMC Health Serv Res. 2024 Mar 13;24:331. doi: 10.1186/s12913-024-10839-x (PMC10935870; doi:10.1186/s12913-024-10839-x)
Supplement: Supplementary file 2 — Supplementary Material 2. [file 12913_2024_10839_MOESM2_ESM.docx]

**Additional file 2**

Summarised description of the main analysis steps in the overview

| **Step** | **Description** |
| --- | --- |
| **1** | **International data set:**   - Supplementation of the Swiss data set with Austrian data sets (2017-2019). In total data of 248 hospitals and 69,645 cases. - Exclusion of cases: Non-participating cases (*n* = 16,690); cases younger than 18 years (*n* = 1,797); cases with missing information regarding outcome or risk variables (*n* = 2,925); cases from non-acute care hospitals (*n* = 1,938); cases from ward types that were only included in the measurements in one country (*n* = 604) *Filter used: G_pat_part=1 AND D_Age>17 AND RANGE(Fall_Prev_30days_inst,0,1) AND RANGE(Fall_Prev_12months,0,1) AND RANGE(Fall_drugs,0,1) AND (InstitutionForm_Type_Inst=1 OR (InstitutionForm_Type_Inst=2 OR InstitutionForm_Type_Inst=3 OR InstitutionForm_Type_Inst=4 AND NOT InstitutionForm_TypeSub_Inst = 5) OR (InstitutionForm_Type_Inst=4 AND InstitutionForm_TypeSub_Inst=43) OR InstitutionForm_Type_Inst=6) AND (RANGE(Type_Ward,0,8) OR RANGE(Type_Ward,16,600) OR RANGE(Type_Ward,602,9999))* |
| **2** | **Standardised data set:** Exclusion of hospitals with fewer than 50 participants over the three measurement years (*n_hospitals_* = 72; *n_cases_* = 1,707) |
| **3** | **Sample Description:**   - Description of hospitals and patient-related fall risk factors - Comparison of risk factors between Switzerland and Austria using Pearson's chi-square test.   **Illustration of a non-risk-adjusted country comparison of inpatient fall rates:**   - Two-level random intercept logistic regression model (hospitals modelled as a random effect and country as a fixed effect). |
| **Research objective #1:** To investigate whether the patient-related fall risk variables included in the inpatient fall risk adjustment model by Bernet et al. [28] show a constant risk relationship with outcome by not interacting with country, thus allowing the model to be used for a risk-adjusted comparison of the odds of falling in hospital in Switzerland and Austria. | |
| **4** | **Detection of interaction effects (Model 1):**   - Two-level random intercept logistic regression model (hospitals modelled as a random effect and patient-related fall risk factors [see Table 1] as well as interaction terms between risk factors and the country as fixed effects). |
| **5** | **Capture the main interaction effects (Model 2):**   - Reduced two-level random intercept logistic regression model (hospitals modelled as a random effect and patient-related fall risk factors [see Table 1] as well as only main interaction terms as fixed effects). Decision criteria for main interaction terms:   1. 2x2 interaction terms: *P*-value of interaction effect < 0.16 in Model 1   2. Interaction term “Age*country” or “CDS*country”: AIC value of model 1 < than AIC value of model 1 without interaction term “Age*country” respectively “CDS*country” |
| **6** | **Performance comparison of the inpatient fall risk adjustment model by country:**   - Application of a two-level random intercept logistic regression model (hospitals modelled as a random effect and patient-related fall risk factors [see Table 1] as fixed effects) to Swiss and Austrian data separately. - Comparison of the discrimination performance of the two models using receiver operating characteristic (ROC) curve analysis. |
| **Research objective #2:** To investigate whether the odds of falling in hospital in Switzerland and Austria differ after risk adjustment. | |
| **7** | **Illustration of a risk-adjusted country comparison of inpatient fall rates:**   - Application of a two-level random intercept logistic regression model (hospitals modelled as a random effect and patient-related fall risk factors [see Table 1] as fixed effects) |
