# Supplementary material for: Comparing risk-adjusted inpatient fall rates internationally: validation of a risk-adjustment model using multicentre cross-sectional data from hospitals in Switzerland and Austria
Source: BMC Health Serv Res. 2024 Mar 13;24:331. doi: 10.1186/s12913-024-10839-x (PMC10935870; doi:10.1186/s12913-024-10839-x)
Supplement: Supplementary file 3 — Supplementary Material 3. [file 12913_2024_10839_MOESM3_ESM.docx]

**Additional file 3**

A graphic overview of patient-related fall risk factors in Switzerland and Austria in comparison
